# Association between systemic immune-inflammation index at admission and post-stroke depression in patients with acute ischemic stroke

**DOI:** 10.3389/fneur.2025.1686621

**Published:** 2025-12-12

**Authors:** Xiaohang Su, Fengtian Chi, Jiulin You, Jueyu Zhao, Sanqi Wang, Xinyu Zhou, Xin Li

**Affiliations:** Department of Neurology, The Affiliated Hospital of Qingdao University, Qingdao, China

**Keywords:** acute ischemic stroke, post-stroke depression, systemic immune-inflammation index, first-ever, biomarker

## Abstract

**Background:**

Post-stroke depression (PSD) is one of the most common neuropsychiatric complications among stroke survivors, with a substantial impact on functional recovery and quality of life. This study aimed to investigate the association between the systemic immune-inflammation index (SII) at admission and the occurrence of PSD in patients with acute ischemic stroke (AIS).

**Methods:**

We prospectively enrolled 318 consecutive patients with first-ever AIS admitted to our hospital between August 2024 and March 2025. Venous blood samples were collected at admission, and SII was calculated as neutrophil count × platelet count/lymphocyte count. At 3 months post-stroke, depressive symptoms were assessed using the 17-item Hamilton Depression Rating Scale (HAMD-17). Patients with a HAMD-17 score >7 were diagnosed with PSD and categorized accordingly into PSD and non-PSD groups.

**Results:**

At the 3-month follow-up, 98 patients (30.82%) were diagnosed with PSD. Compared with the non-PSD group, patients in the PSD group had significantly higher SII values [658.66 (468.73–958.90) vs. 476.71 (362.73–646.83), *p* < 0.001]. In multivariate logistic regression analysis, after adjusting for potential confounders, patients in the highest SII tertile had a significantly increased risk of developing PSD compared with those in the lowest tertile (OR = 3.502, 95% CI: 1.582–7.752, *p* = 0.002). Receiver operating characteristic (ROC) curve analysis identified an optimal SII cut-off value of 602.503 for predicting PSD, with a sensitivity of 0.582, a specificity of 0.700, and an area under the curve (AUC) of 0.659 (95% CI: 0.592–0.726, *p* < 0.001).

**Conclusion:**

Elevated SII levels at admission are positively associated with the development of PSD in AIS patients, suggesting that SII may serve as a valuable inflammatory biomarker for early identification of patients at high risk for PSD.

## 1 Introduction

According to the 2019 Global Burden of Disease (GBD) study, stroke remains the second leading cause of death worldwide (1). It is well established that stroke can lead to various complications, including cognitive impairment, physical disability, and psychiatric disorders (2–4). Among these, post-stroke depression (PSD) is one of the most common neuropsychiatric complications (5). A meta-analysis by Hackett et al. encompassing 51 studies reported that approximately one-third of stroke survivors develop PSD (6). Accumulating evidence indicates that PSD is closely associated with poor functional outcomes, increased mortality, and reduced quality of life (7, 8). Therefore, early identification and diagnosis of PSD are crucial for reducing stroke-related complications and improving patient prognosis.

Previous studies have demonstrated that pro-inflammatory cytokines play a crucial role in the inflammatory response of acute ischemic stroke (AIS) and depression, and have been confirmed to be closely associated with the development of PSD (9, 10). It has been reported that within 1 year after stroke onset, inflammatory markers such as interleukin-6 (IL-6), interleukin-10 (IL-10), and tumor necrosis factor-alpha (TNF-*α*) are significantly elevated, with notable increases in the IL-6/IL-10 and TNF-*α*/IL-10 ratios (11). Furthermore, a longitudinal study indicated that elevated levels of TNF-α and interleukin-1 beta (IL-1β) within 2 weeks post-stroke are strongly correlated with the occurrence of PSD (12). Additionally, a six-month follow-up study found that increased serum high-sensitivity C-reactive protein (hs-CRP) levels at admission were also associated with PSD (13). Collectively, these findings suggest that systemic inflammation plays a pivotal role in the pathophysiology of PSD.

The systemic immune-inflammation index (SII) is a novel inflammatory marker derived from a composite calculation of neutrophil, lymphocyte, and platelet count (14). The concept of SII was first proposed by Hu et al. (14), initially for assessing the prognosis of patients with solid tumors and coronary artery disease (15, 16), and has since been widely recognized as an effective indicator of the systemic inflammatory status (17). In recent years, increasing attention has been directed toward the relationship between SII and AIS. Meta-analyses have demonstrated that elevated SII levels at admission are associated with unfavorable functional outcomes and higher mortality in AIS patients (18, 19). In addition, SII has also been shown to predict the risk of hemorrhagic transformation following AIS (20). Notably, emerging evidence suggests a link between SII and emotion-related disorders. A longitudinal study by Mazza et al. (21) reported a significant positive correlation between SII and depressive and anxiety symptoms among COVID-19 survivors. Other studies have found that elevated SII levels are associated with poorer clinical prognosis in patients with major depressive disorder (22), suggesting that SII may serve as a marker of low-grade inflammation in mood disorders. Although existing evidence suggests a potential association between SII and PSD, prospective data remain scarce, and the extent to which stroke lesion location may influence this relationship is not well understood. Therefore, this prospective cohort study investigated the relationship between admission SII and PSD in first-ever AIS patients. We additionally explored whether lesion location modifies the SII–PSD association and evaluated the predictive performance of SII using tertile analyses and receiver operating characteristic (ROC) curves. We hypothesized that elevated SII at admission is independently associated with an increased risk of PSD and may serve as a valuable clinical biomarker for early risk stratification.

## 2 Materials and methods

This study was approved by the Ethics Committee of the Affiliated Hospital of Qingdao University, and informed consent was obtained from all patient or their family members. Consecutive patients diagnosed with AIS and admitted to the Affiliated Hospital of Qingdao University between August 2024 and March 2025 were prospectively enrolled.

**Figure 1:**
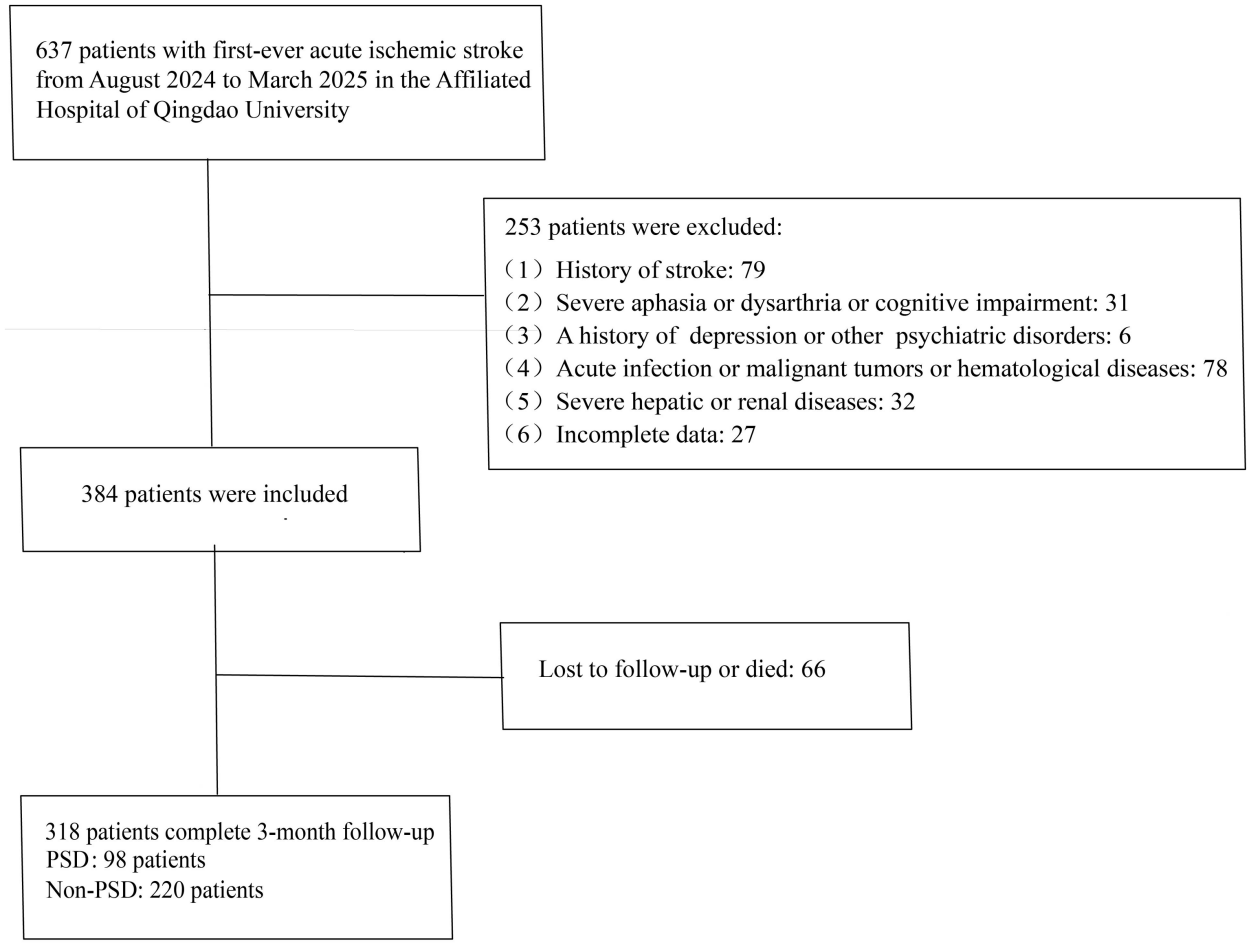
Flow chart for patients’ selection.

The inclusion criteria were as follows: 1. age ≥18 years; 2. admission within 7 days of symptom onset; 3. confirmation of ischemic cerebral infarction by cranial computed tomography (CT) or magnetic resonance imaging (MRI), meeting the diagnostic criteria outlined in the Chinese Guidelines for the Diagnosis and Treatment of Acute Ischemic Stroke (23), and identified as a first-ever stroke; 4. ability to complete follow-up with complete clinical data available. Exclusion criteria included: 1. history of stroke; 2. severe aphasia, dysarthria, disturbance of consciousness, or cognitive impairment; 3. prior history of depression or other psychiatric disorders; 4. concomitant acute infection, malignancy, or hematological disorders; 5. severe hepatic or renal dysfunction. A total of 318 patients met the eligibility criteria and were included in the final analysis (Figure 1).

### 2.1 Data collection

Baseline characteristics were collected for all enrolled patients, including age, sex, marital status, educational level, and vascular risk factors (history of smoking, alcohol consumption, hypertension, diabetes mellitus, hyperlipidemia, coronary heart disease, and atrial fibrillation). All patients underwent cranial CT or MRI within 48 h of admission to identify the location of cerebral infarction lesions. The etiology of stroke was classified according to the Trial of Org 10,172 in Acute Stroke Treatment (TOAST) criteria (24). Venous blood samples were obtained to measure peripheral blood cell counts, fibrinogen (FIB), uric acid (UA), low-density lipoprotein cholesterol (LDL-C), high-density lipoprotein cholesterol (HDL-C), total cholesterol (TC), triglycerides (TG), urea (Ur), creatinine (Cr), fasting blood glucose (FBG), alanine aminotransferase (ALT), aspartate aminotransferase (AST), and homocysteine (HCY) levels. The SII was calculated as: neutrophil count × platelet count/lymphocyte count. Within 24 h of admission, stroke severity was assessed by a neurologist using the National Institutes of Health Stroke Scale (NIHSS). Functional outcomes at discharge were evaluated using the Barthel Index (BI) and the modified Rankin Scale (mRS).

### 2.2 Psychological measurement

At 3 months post-stroke, patients were followed up either face-to-face or via telephone. Depressive symptoms were assessed using the 17-item Hamilton Depression Rating Scale (HAMD-17) (25), with a score greater than 7 indicating a diagnosis of PSD (26). All neuropsychological assessments were conducted by neurologists who had received specialized training.

### 2.3 Statistical analysis

All statistical analyses were performed using SPSS version 27.0. The Kolmogorov–Smirnov test was used to assess the normality of continuous variables. Normally distributed continuous variables are presented as mean ± standard deviation, while non-normally distributed variables are expressed as median with interquartile range. Categorical variables are presented as percentages(%). Comparisons between groups for normally distributed variables were conducted using independent-samples t tests, whereas non-normally distributed variables were compared using the Mann–Whitney U test. Categorical variables were analyzed using Pearson’s chi-square test or Fisher’s exact test, as appropriate. Differences across SII strata were evaluated using the Kruskal-Wallis test or one-way analysis of variance (ANOVA). After adjusting for conventional confounders and major baseline variables associated with PSD identified in univariate logistic regression, multivariate logistic regression was performed to calculate odds ratios (ORs) and 95% confidence intervals (CIs) for the risk of PSD. ROC curve analysis was used to determine the optimal cut-off value of admission SII levels for predicting PSD at 3-month follow-up, and the area under the curve (AUC) was calculated to assess predictive accuracy. All statistical tests were two-sided, and a *p*-value < 0.05 was considered statistically significant.

## 3 Results

### 3.1 Baseline characteristics of patients in the PSD group and the non-PSD group

A total of 637 patients with AIS admitted to the Department of Neurology at Qingdao University Affiliated Hospital between August 2024 and March 2025 were screened. Among them, 384 patients met the inclusion criteria, while 253 met the exclusion criteria. Additionally, 66 patients were excluded due to loss to follow-up or death. Ultimately, 318 patients were included in the analysis. During the 3-month follow-up period, 98 patients (30.82%) were diagnosed with PSD.

Table 1 presents demographic characteristics, vascular risk factors, laboratory parameters, and neuropsychological assessment results stratified by PSD status. Compared to the non-PSD group, patients with PSD showed significantly higher neutrophil count (*p* = 0.006), platelet count (*p* < 0.001), HCY levels (*p* = 0.017), SII levels (*p* < 0.001), NIHSS score (*p* < 0.001), and mRS score (*p* < 0.001), while BI score (*p* < 0.001) and lymphocyte count (*p* = 0.043) were significantly lower. Furthermore, lesion locations in the temporal lobe (*p* = 0.015) and basal ganglia (*p* = 0.010) were significantly associated with an increased risk of PSD.

**Table 1:** Clinical and demographic characteristics of patients with PSD and non-PSD.

Variables	Total(*n* = 318)	Non-PSD patients(*n* = 220)	PSD patients(*n* = 98)	*P*-value
Age (years)	63(57–71)	64(58–72)	61(56–70)	0.099
Gender, *n*(%)				0.415
Female	101(31.76%)	73(33.18%)	28(28.57%)	
Male	217(68.24%)	147(66.82%)	70(71.43%)	
Marital status, *n*(%)				0.180
Unmarried	6(1.89%)	5 (2.27%)	1(1.02%)	
Married	285(89.62%)	197(89.55%)	88(89.80%)	
Divorced	8(2.52%)	3(1.36%)	5(5.10%)	
Widowed	19(5.97%)	15(6.82%)	4(4.08%)	
Education, *n*(%)				0.620
Primary school and below	120(37.74%)	85(38.64%)	35(35.71%)	
Junior high school and above	198(62.26%)	135(61.36%)	63(64.59%)	
Lesion location, *n*(%)				
Frontal lobe	80(25.16%)	49(22.27%)	31(31.63%)	0.076
Parietal lobe	72(22.64%)	50(22.73%)	22(22.45%)	0.956
Temporal lobe	40(12.58%)	21(9.55%)	19(19.39%)	0.015
Occipital lobe	57(17.92%)	35(15.91%)	22(22.45%)	0.160
Basal ganglia	107(33.65%)	64(29.09%)	43(43.88%)	0.010
Thalamus	35(11.01%)	28(12.73%)	7(7.14%)	0.142
Cerebellum	30(9.43%)	20(9.09%)	10(10.20%)	0.754
Brain stem	73(22.96%)	49(22.27%)	24(24.49%)	0.664
Lateralization, *n*(%)				
Left hemisphere	138(43.40%)	99(45.00%)	39(39.80%)	0.387
Right hemisphere	142(44.65%)	95(43.18%)	47(47.96%)	0.429
Bilateral hemisphere	38(11.95%)	26(11.82%)	12(12.24%)	0.914
TOAST subtypes, *n*(%)				0.170
LAA	134(42.27%)	84(38.36%)	50(51.02%)	
SAO	137(43.22%)	103(47.03%)	34(34.69%)	
CE	11(3.47%)	6(2.74%)	5(5.10%)	
SOE	9(2.84%)	6(2.74%)	3(3.06%)	
SUE	26(8.20%)	20(9.13%)	6(6.12%)	
Vascular risk factors, *n*(%)				
Current smoking	141(44.34%)	96(43.64%)	45(45.92%)	0.705
Current drinking	150(47.17%)	98(44.55%)	52(53.06%)	0.160
Hypertension	231(72.64%)	165(75.00%)	66(67.35%)	0.157
Diabetes mellitus	93(29.25%)	61(27.73%)	32(32.65%)	0.373
Hyperlipidaemia	124(38.99%)	81(36.82%)	43(43.88%)	0.233
Coronary heart disease	27(8.49%)	18(8.18%)	9(9.18%)	0.767
Atrial fibrillation	14(4.40%)	9(4.09%)	5(5.10%)	0.685
Laboratory tests				
WBC (10^9^/L)	6.77(5.78–8.12)	6.55(5.64–8.00)	7.23(5.95–8.33)	0.076
Neutrophil (10^9^/L)	4.37(3.50–5.34)	4.26(3.38–5.23)	4.78(3.80–5.83)	0.006
Lymphocyte (10^9^/L)	1.74(1.40–2.19)	1.79(1.47–2.25)	1.64(1.24–2.07)	0.043
Monocyte (10^9^/L)	0.38(0.32–0.46)	0.39(0.32–0.47)	0.38(0.32–0.46)	0.508
Platelet (10^9^/L)	217.00(187.00–256.00)	209.00(183.25–249.00)	235.50(196.50–279.25)	<0.001
SII	520.24(384.74–740.37)	476.71(362.73–646.83)	658.66(468.73–958.90)	<0.001
FIB (g/L)	3.04(2.70–3.39)	3.03(2.69–3.30)	3.14(2.71–3.56)	0.099
UA (umol/L)	298.30 ± 73.52	283.02 ± 74.22	283.94 ± 72.28	0.795
HDL-C (mmol/L)	3.83(1.61–4.72)	1.17(0.97–1.65)	1.18(0.96–2.68)	0.574
LDL-C (mmol/L)	2.83(2.06–3.81)	2.81(2.09–3.78)	2.93(1.93–4.17)	0.477
TC (mmol/L)	3.83(1.61–4.72)	3.87(2.48–4.72)	3.62(1.22–4.77)	0.168
TG (mmol/L)	1.65(1.08–3.76)	1.50(1.04–3.16)	2.06(1.13–4.15)	0.051
FBG (mmol/L)	4.99(3.42–6.00)	5.00(3.83–5.85)	4. 90(2.96–6.36)	0.587
Urea (mmol/L)	6.10(5.10–7.30)	6.20(5.00–7.48)	5.90(5.10–7.15)	0.392
Cr (umol/L)	59.45(50.80–69.20)	59.90(50.80–69.35)	57.80(49.08–70.13)	0.512
ALT (U/L)	21.00(16.38–27.00)	21.00(16.00–26.00)	22.40(17.00–30.00)	0.172
AST (U/L)	24.00(20.00–27.00)	24.00(19.55–28.00)	23.00(20.00–27.00)	0.584
HCY (umol/L)	10.20(8.00–12.38)	9.75(7.80–12.10)	11.00(8.80–13.33)	0.017
Neuropsychological function				
NIHSS score	2(0–5)	1(0–3)	4(2–7)	<0.001
BI score	75(60–90)	75(65–90)	65(50–84)	<0.001
mRS score	1(0–2)	1(0–1)	2(1–4)	<0.001
HAMD score	3(2–9)	3(2–4)	11(9–17)	<0.001

PSD, post-stroke depression; IQR, interquartile range; SD, standard deviation; TOAST: trial of organization in acute stroke treatment 10,172 in acute stroke treatment; LAA, large-artery atherosclerosis; SAO, small-artery occlusion; CE, cardioembolism; SOE, stroke of other determined etiology; SUE, stroke of undetermined etiology; WBC, white blood cell; SII, systemic immune–inflammation index; FIB, fibrinogen; UA, uric acid; HDL-C, high-density lipoprotein cholesterol; LDL-C, low-density lipoprotein cholesterol; TC, total cholesterol; TG, triglyceride; FBG, fasting blood glucose; Cr, creatinine; ALT, alanine aminotransferase; AST, aspartate aminotransferase; HCY, homocysteine; NIHSS, National Institutes of Health Stroke Scale; BI, Barthel Index; mRS, modified Rankin Scale; HAMD, Hamilton depression scale.

### 3.2 Baseline characteristics of all patients in SII tertiles

Table 2 shows that patients were stratified into three subgroups according to the tertiles of SII level: T1 (<414.13), T2 (414.13–645.56), and T3 (>645.56). No statistically significant differences were observed among the three groups in terms of age, sex, marital status, educational level, infarct location, or vascular risk factors (*p* > 0.05). With increasing SII levels, white blood cell count, neutrophil count, and platelet count increased significantly (*p* < 0.001), whereas lymphocyte count decreased significantly (p < 0.001). The FIB level in the T3 group was significantly higher than that in the other two groups (*p* = 0.040). Differences in UA, lipid profiles (HDL-C, LDL-C, TC, TG), and liver/kidney function indicators (ALT, AST, Cr) were not statistically significant among the groups. Neurological and psychological function assessments revealed that patients in the T3 group had more severe neurological deficits (*p* = 0.002) and more pronounced depressive symptoms (*p* = 0.005). However, there were no significant differences in BI score or mRS score among the groups (*p* > 0.05).

**Table 2:** Baseline characteristics of patients with AIS according to SII tertiles.

Variables	SII tertiles			*P*-value
	T1 (<414.13, *n* = 106)	T2 (414.13–645.56, *n* = 106)	T3 (>645.56, *n* = 106)	
Age (years)	63(57–71)	63(58–71)	63(57–72)	0.895
Gender, *n*(%)				0.828
Female	36(33.96%)	32(30.19%)	33(31.13%)	
Male	70(66.04%)	74(69.81%)	73(68.87%)	
Marital status, n(%)				0.327
Unmarried	2(1.89%)	3(2.83%)	1(0.94%)	
Married	96(90.57%)	95(89.62%)	94(88.68%)	
Divorced	8(7.55%)	5(4.72%)	6(5.66%)	
Widowed	0(0.00%)	3(2.83%)	5(4.72%)	
Education, *n*(%)				0.852
Primary school and below	40(37.74%)	38(35.85%)	42(39.62%)	
Junior high school and above	66(62.26%)	68(64.15%)	64(60.38%)	
Lesion location, *n*(%)				
Frontal lobe	25(23.58%)	30(28.30%)	25(23.58%)	0.659
Parietal lobe	21(19.81%)	28(26.42%)	23(21.70%)	0.496
Temporal lobe	11(10.38%)	13(12.26%)	16(15.09%)	0.581
Occipital lobe	13(12.26%)	19(17.92%)	25(23.58%)	0.099
Basal ganglia	41(38.68%)	30(28.30%)	36(33.96%)	0.278
Thalamus	12(11.32%)	8(7.55%)	15(14.15%)	0.305
Cerebellum	5(4.72%)	14(13.21%)	11(10.38%)	0.098
Brain stem	22(20.75%)	23(21.70%)	28(26.42%)	0.576
Lateralization, *n*(%)				
Left hemisphere	49(46.23%)	44(41.51%)	45(42.45%)	0.764
Right hemisphere	48(45.28%)	45(42.45%)	49(46.23%)	0.848
Bilateral hemisphere	9(8.49%)	17(16.04%)	12(11.32%)	0.231
TOAST subtypes, *n*(%)				0.515
LAA	39(36.79%)	50(47.17%)	45(42.45%)	
SAO	52(49.06%)	40(37.74%)	45(42.45%)	
CE	5(4.72%)	1(0.94%)	5(4.72%)	
SOE	3(2.83%)	3(2.83%)	3(2.83%)	
SUE	7(6.60%)	11(10.38%)	8(7.55%)	
Vascular risk factors, *n*(%)				
Current smoking	43(40.57%)	53(50.00%)	45(42.45%)	0.343
Current drinking	48(45.28%)	53(50.00%)	49(46.23%)	0.767
Hypertension	73(68.87%)	79(74.53%)	79(74.53%)	0.566
Diabetes mellitus	29(27.36%)	35(33.02%)	29(27.36%)	0.579
Hyperlipidaemia	33(31.13%)	43(40.57%)	48(45.28%)	0.099
Coronary heart disease	10(9.43%)	6(5.66%)	11(10.38%)	0.427
Atrial fibrillation	7(6.60%)	1(0.94%)	6(5.66%)	0.101
Laboratory tests				
WBC (10^9^/L)	5.94 (5.20–7.14)	7.03 (6.03–7.94)	7.55(6.26–8.89)	<0.001
Neutrophil (10^9^/L)	3.42(2.89–4.04)	4.53(3.87–5.23)	5.50(4.39–6.84)	<0.001
Lymphocyte (10^9^/L)	2.03(1.72–2.65)	1.92(1.48–2.32)	1.39(1.11–1.68)	<0.001
Monocyte (10^9^/L)	0.36(0.31–0.45)	0.41(0.35–0.49)	0.37(0.30–0.46)	0.003
Platelet (10^9^/L)	199.00(166.75–227.75)	220.50(190.75–256.00)	249.00(200.25–291.50)	<0.001
SII	332.85(268.14–384.74)	520.24(471.33–570.36)	943.03(740.37–1295.35)	<0.001
FIB (g/L)	2.99(2.70–3.29)	3.02(2.60–3.32)	3.17(2.79–3.57)	0.040
UA (umol/L)	287.81 ± 71.27	286. 12 ± 78.27	275.97 ± 70.92	0.422
HDL-C (mmol/L)	1.18(0.98–2.26)	1.17(0.96–2.14)	1.18(0.94–1.87)	0.773
LDL-C (mmol/L)	2.87(1.88–3.86)	2.85(2.12–3.74)	2.82(2.14–3.95)	0.678
TC (mmol/L)	3.69(1.32–4.71)	4.01(2.06–4.69)	3.77(1.61–4.77)	0.689
TG (mmol/L)	1.69(1.13–3.92)	1.70(1.11–3.90)	1.49(0.98–3.43)	0.460
FBG (mmol/L)	5.07(3.06–5.82)	4.99(3.79–6.24)	4.86(3.27–6.23)	0.776
Urea (mmol/L)	6.30(5.00–7.43)	5.95(5.10–7.60)	5.90(4.95–7.20)	0.429
Cr (umol/L)	59.90(50.80–69.68)	60.90(51.45–69.35)	58.00(49.90–69.05)	0.745
ALT (U/L)	21.50(17.00–27.25)	21.00(16.00–27.25)	21.00(16.00–27.00)	0.972
AST (U/L)	25.00(21.00–28.00)	24.00(19.00–27.25)	22.00(19.00–27.00)	0.211
HCY (umol/L)	9.65(7.88–12.03)	10.35(8.50–11.97)	10.80(8.03–13.65)	0.134
Neuropsychological function				
NIHSS score	1(0–3)	2(0–4)	3(0–6)	0.002
BI score	78(65–90)	75(55–90)	70(55–85)	0.118
mRS score	1(0–1)	1(0–1)	1(0–2)	0.130
HAMD score	3(2–6)	3(2–8)	7(2–10)	0.005

IQR, interquartile range; SD, standard deviation; TOAST: trial of organization in acute stroke treatment 10,172 in acute stroke treatment; LAA, large-artery atherosclerosis; SAO, small-artery occlusion; CE, cardioembolism; SOE, stroke of other determined etiology; SUE, stroke of undetermined etiology; WBC, white blood cell; SII, systemic immune–inflammation index; FIB, fibrinogen; UA, uric acid; HDL-C, high-density lipoprotein cholesterol; LDL-C, low-density lipoprotein cholesterol; TC, total cholesterol; TG, triglyceride; FBG, fasting blood glucose; Cr, creatinine; ALT, alanine aminotransferase; AST, aspartate aminotransferase; HCY, homocysteine; NIHSS, National Institutes of Health Stroke Scale; BI, Barthel Index; mRS, modified Rankin Scale; HAMD, Hamilton depression scale.

### 3.3 Association between the level of SII and PSD

**Figure 2:**
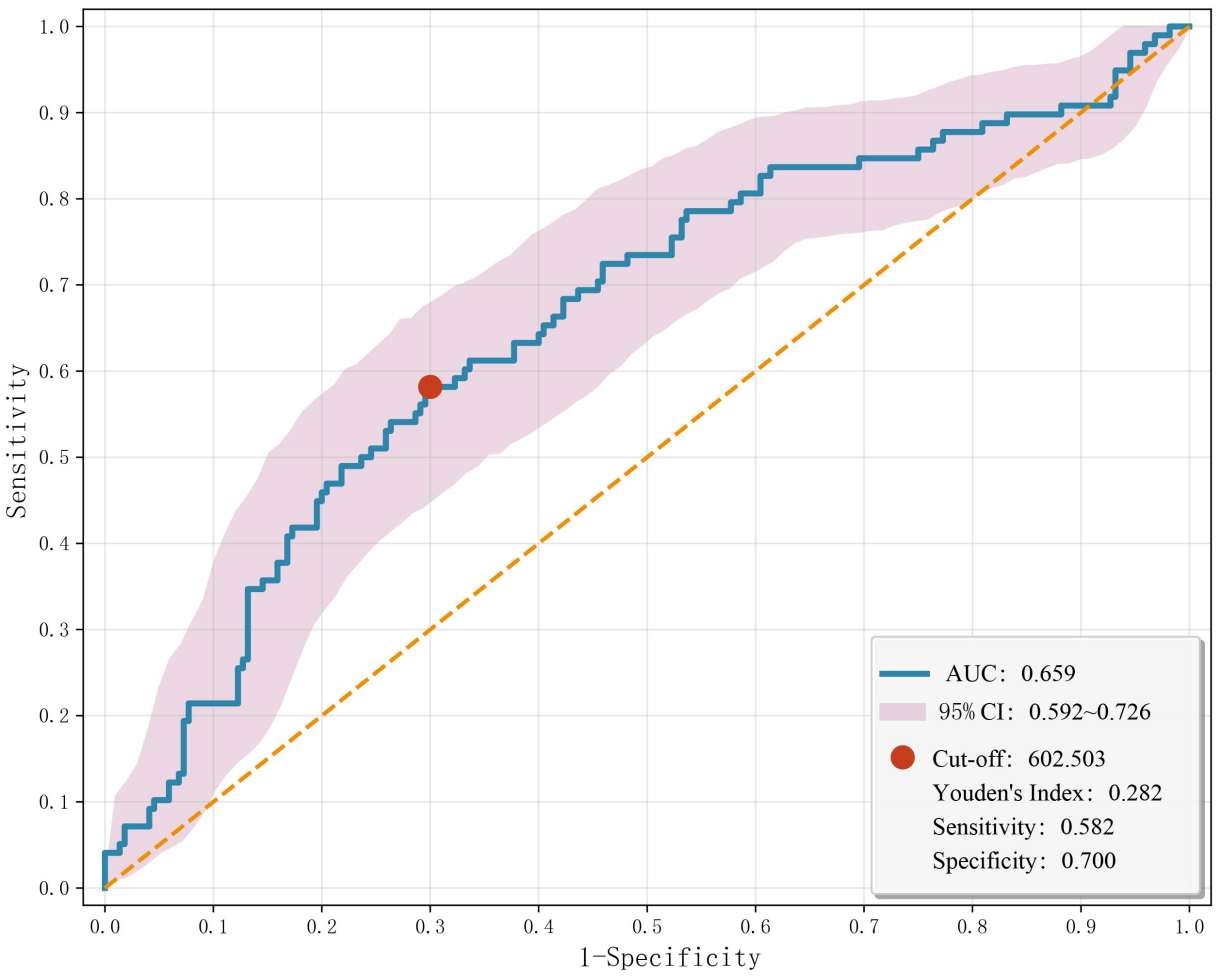
The ROC curve analysis for SII to predict PSD in AIS patients at 3 months.

A statistically significant difference in the distribution of SII tertiles was observed between the PSD and non-PSD groups (χ^2^ = 22.15, *p* < 0.001). In the PSD group, the proportion of patients in the lowest tertile was significantly lower (p < 0.001), whereas the proportion in the highest tertile was significantly higher (p < 0.001). Specifically, the numbers of PSD patients in the low, middle, and high SII tertiles were 19 (19.39%), 29 (29.59%), and 50 (51.02%), respectively (Table 3). In Table 4, all patients were analyzed as a whole, with the occurrence of PSD as the dependent variable and the lowest SII tertile as the reference category. Logistic regression analyses were performed to evaluate the association between SII and PSD. In the unadjusted model, patients in the highest SII tertile had a significantly higher risk of PSD compared with those in the lowest tertile (unadjusted: OR = 4.088, 95% CI: 2.186–7.645, *p* < 0.001). After sequential adjustment for potential confounders—including age, sex, marital status, educational level, vascular risk factors (smoking, alcohol consumption, hypertension, diabetes mellitus, hyperlipidemia, coronary heart disease, and atrial fibrillation), NIHSS score, Barthel Index, mRS score, lesion location (temporal lobe and basal ganglia), FIB, and HCY—the highest SII tertile remained independently and significantly associated with an increased risk of PSD (Model 1: OR = 3.895, 95% CI = 2.064–7.350, *p* < 0.001; Model 2: OR = 4.199, 95% CI = 2.104–7.689, p < 0.001; Model 3: OR = 3.502, 95% CI = 1.582–7.752, *p* = 0.002). The predictive performance of SII for PSD was evaluated using ROC curve analysis (Figure 2). The AUC was 0.659 (95% CI: 0.592–0.726, *p* < 0.001), indicating a moderate discriminatory ability. The optimal cut-off value was 602.503, yielding a sensitivity of 0.582, a specificity of 0.700, and a Youden index of 0.282. These findings suggest that SII has some predictive value for PSD, although its diagnostic performance is relatively limited.

**Table 3:** SII tertiles of patients.

Variables	Depression(*n* = 98)	Non-depression(*n* = 220)	χ^2^	*P*-value
SII			22.15	<0.001
Tertile 1	19(19.39%)	87(39.55%)	12.40	<0.001
Tertile 2	29(29.59%)	77(35.00%)	0.89	0.345
Tertile 3	50(51.02%)	56(25.45%)	19.94	<0.001

**Table 4:** Multivariate logistic regression analysis model of SII and PSD.

	Tertile	OR^a^	95%CI	*P*-value
Unadjusted	Middle	1.725	0.896–3.319	0.103
	Highest	4.088	2.186–7.645	<0.001
Model 1^b^	Middle	1.659	0.855–3.220	0.135
	Highest	3.895	2.064–7.350	<0.001
Model 2^c^	Middle	1.690	0.858–3.327	0.129
	Highest	4.199	2.104–7.689	<0.001
Model 3^d^	Middle	1.607	0.708–3.647	0.257
	Highest	3.502	1.582–7.752	0.002

OR, odds ratio; CI, confidence interval; PSD, post-stroke depression; SII, systemic immune–inflammation index.

a Reference OR (1.000) is the lowest tertile of SII for PSD.

b Model 1: adjusted for age, sex, marital status, education, current smoking, and current alcohol drinking.

c Model 2: adjusted for covariates from Model 1 and further adjusted for medical history (hypertension, diabetes mellitus, hyperlipidemia, coronary heart disease, atrial fibrillation).

d Model 3: adjusted for covariates from Model 2 and further adjusted for baseline NIHSS score, Barthel Index score, mRS score, Lesion location (Temporal lobe, Basal ganglia), FIB, and HCY.

## 4 Discussion

The findings of this study demonstrate that elevated SII levels at admission are significantly associated with the development of depressive symptoms within 3 months after stroke in patients experiencing their first-ever AIS. Multivariable analyses further confirmed that, after adjusting for major confounding factors, patients in the highest SII tertile had a 3.502-fold greater risk of PSD compared with those in the lowest tertile. Approximately 30.82% of AIS patients developed depressive symptoms at 3 months, a prevalence consistent with previous reports (6). Moreover, patients with PSD exhibited more severe neurological deficits and poorer functional outcomes than those without PSD. Prior meta-analytic evidence has highlighted that PSD is strongly associated with decreased quality of life, higher disability rates, and increased mortality (27), highlighting the importance of early identification and intervention. Collectively, our results reinforce the critical role of peripheral inflammation in the development of PSD and suggest that SII—an easily accessible and cost-effective biomarker—may be valuable for early risk stratification.

Importantly, unlike previous studies that have mainly focused on associations between inflammatory markers and PSD, this prospective study is the first to systematically evaluate the potential modifying role of stroke lesion location in the SII–PSD relationship. Our findings showed that temporal lobe and basal ganglia lesions were significantly associated with PSD, suggesting that damage to these specific brain regions may contribute to its underlying pathophysiological mechanisms. This observation is consistent with the report by Zhang et al., who identified temporal cortico-subcortical involvement as a correlate of PSD (28). Likewise, Chatterjee et al. confirmed a close relationship between basal ganglia lesions and affective symptoms, including depression (29). Notably, even after adjusting for lesion locations in multivariable regression models, patients in the highest SII tertile continued to have a markedly increased risk of PSD (OR = 3.502, 95% CI: 1.582–7.752, *p* = 0.002). These findings suggest that the relationship between systemic inflammatory burden and PSD is not fully explained by lesion location alone, and that inflammatory responses may interact with structural brain injury, influencing neurotransmitter pathways and emotion-regulation circuits to facilitate the development of PSD. This study therefore provides novel clinical evidence supporting a potential interplay between lesion topography and systemic inflammation in the pathophysiology of PSD.

A growing body of evidence supports the role of inflammation in the onset and progression of depression. In a multicenter cross-sectional study of 338 hospitalized patients with tuberculosis, nearly half exhibited anxiety or depressive symptoms. Compared with asymptomatic individuals, these patients demonstrated poorer cellular immune function and heightened inflammatory responses, with elevated SII levels significantly associated with emotional symptoms (*p* < 0.05) (30). Similarly, a cohort study of 2,566 patients with diabetes mellitus identified elevated SII as an independent risk factor for depression (OR = 1.347, 95% CI: 1.031–1.760, *p* = 0.02), a finding further supported by propensity score–matched analyses (31). Another cross-sectional study showed that patients with major depressive disorder had significantly higher SII levels than healthy controls, particularly in relation to systemic inflammation and coronary heart disease risk (32). Moreover, in a follow-up study of 109 COVID-19 survivors, baseline NLR and SII were positively correlated with depressive and anxiety symptom scores at 15 days post-discharge (33). Collectively, these studies indicate that elevated SII is consistently associated with an increased risk of depression across diverse clinical populations, reinforcing the role of peripheral inflammation in depressive psychopathology.

Inflammatory cell infiltration and the sustained upregulation of pro-inflammatory mediators play central roles in the pathophysiological processes of AIS and its associated emotional disturbances. Neutrophils are among the earliest peripheral immune cells recruited to infarcted brain tissue during acute stroke (34). Through the release of matrix metalloproteinase-9 (MMP-9), cytokines (e.g., IL-6), chemokines (e.g., MCP-1), proteolytic enzymes, and reactive oxygen species (35, 36), neutrophils amplify excitotoxic inflammatory responses and disrupt the integrity of the blood–brain barrier (BBB) (37). BBB disruption facilitates the entry of peripheral immune cells and inflammatory mediators into central emotion-regulating circuits—such as the hippocampus, prefrontal cortex, and amygdala—thereby disrupting neural homeostasis and contributing to the development of PSD. Multiple peripheral inflammatory markers (e.g., hs-CRP, ferritin, TNF-*α*, IL-1β, IL-6, IL-18, IFN-*γ*) have been shown to be significantly associated with PSD (38). Platelets also play a crucial role, particularly during early inflammation, by interacting with neutrophils to amplify inflammatory signaling cascades (39). Additionally, platelets serve as the primary peripheral reservoir of serotonin (5-HT). Upon activation in inflammatory states, they release substantial amounts of 5-HT and pro-inflammatory mediators, disturbing neurotransmitter balance and neuroendocrine function and thereby increasing vulnerability to mood disorders (40, 41). Lymphocytes exhibit more complex and subtype-specific functions in AIS. CD4+/CD8 + T cells and natural killer (NK) cells can exacerbate neuroinflammation through the release of neurotoxic cytokines such as interferon-*γ* and IL-17 (42), whereas regulatory T cells (Tregs) exert neuroprotective effects by producing IL-10 and modulating signaling pathways including JAK/STAT, PI3K, and MAPK (43). Metabolic abnormalities in peripheral CD4 + T cells may also induce purine metabolism dysfunction, leading to anxiety, depression, and deficits in social behavior (44). Furthermore, marked lymphopenia within 24 h after stroke indicates pathological stress and immunosuppression, which can activate the hypothalamic–pituitary–adrenal (HPA) axis, disrupt monoamine neurotransmission, and increase the risk of mood disorders (45). Acute stroke-induced inflammation also triggers oxidative stress, neurotransmitter imbalance, and activation of the indoleamine-2,3-dioxygenase (IDO) pathway, resulting in serotonin depletion and promoting depressive symptoms (46). Taken together, peripheral neutrophils, lymphocytes, platelets, and their complex interactions contribute to systemic inflammation following AIS, providing a biological foundation for the role of SII as a predictive marker of PSD.

Previous studies have shown that inflammation-based markers such as NLR and PLR can serve as low-cost biomarkers for predicting PSD (47). As a composite index incorporating neutrophil, lymphocyte, and platelet count, SII may provide a more comprehensive reflection of systemic inflammation and immune homeostasis than either NLR or PLR alone. Higher SII values indicate a heightened inflammatory response accompanied by reduced immunoregulatory capacity. A cross-sectional study demonstrated that patients with PSD had significantly higher SII levels than those without PSD, identifying SII > 547.30 as an independent risk factor (OR = 2.18, 95% CI: 1.274–3.732, *p* = 0.004) (48). Similarly, an NHANES-based cross-sectional analysis reported that each one-unit increase in log₂-SII was associated with an 18% increase in depression prevalence (49). In our study, SII levels were notably higher in the PSD group (658.66 vs. 476.71, *p* < 0.001). ROC analysis yielded an AUC of 0.659, with an optimal cut-off value of 602.503 (sensitivity = 0.582; specificity = 0.700), indicating moderate discriminatory ability. Although the independent predictive value of SII is limited, its low cost, wide availability, and ease of measurement make it a promising addition to routine post-stroke assessments for early identification and targeted follow-up of patients at elevated risk for PSD.

This study has several limitations. First, it was a single-center study with a relatively small sample size, which may have reduced statistical power and limited the generalizability of the findings. Second, the 3-month follow-up period may not adequately capture the dynamic course of PSD, which can fluctuate or manifest later—such as at 6 or 12 months after stroke—thereby potentially weakening the observed associations between inflammatory markers and PSD. Third, patients with impaired consciousness, severe dysarthria, or aphasia were excluded, which may have introduced selection bias, as these individuals typically have more severe neurological deficits and may be at higher risk for PSD. Fourth, loss to follow-up may have resulted in survival bias, since individuals who were lost or deceased may have experienced more severe strokes and exhibited higher baseline SII levels. Fifth, SII was measured only once, which limited the ability to assess temporal changes in inflammation and their relationship to PSD onset. Therefore, larger multicenter cohort studies with extended follow-up durations and longitudinal monitoring of inflammatory markers are needed to validate our findings and further elucidate the causal pathways linking inflammation to PSD.

## 5 Conclusion

In summary, elevated SII levels in the acute phase of AIS are independently associated with an increased risk of PSD, indicating that SII may represent a promising biomarker for clinical application. Future research should aim to develop and validate integrated predictive models that incorporate SII alongside clinical, neuroimaging, and psychosocial risk factors to enhance the accuracy of PSD prediction. Such models may ultimately support more targeted monitoring strategies and facilitate earlier intervention among high-risk patients.

## Data Availability

The original contributions presented in the study are included in the article/supplementary material, further inquiries can be directed to the corresponding author.
